# Shedding and transmission of a live attenuated influenza A virus vaccine in pre-weaned pigs under field conditions

**DOI:** 10.1371/journal.pone.0246690

**Published:** 2021-02-11

**Authors:** Gustavo Lopez Moreno, Jayaveeramuthu Nirmala, Christa Goodell, Marie Culhane, Montserrat Torremorell

**Affiliations:** 1 Department of Veterinary Population Medicine, College of Veterinary Medicine, University of Minnesota, Saint Paul, Minnesota, United States of America; 2 Boehringer Ingelheim Animal Health USA Inc., Duluth, Georgia, United States of America; University of South Dakota, UNITED STATES

## Abstract

Influenza A virus (IAV) is one of the most important respiratory viruses affecting pig health and vaccination is the most common strategy to control influenza infections. In this field study we assessed the onset and duration of shedding of a live attenuated influenza virus (LAIV) vaccine, its ability to transmit to non-vaccinated pigs and whether the LAIV could be aerosolized and detected in the environment. Thirty-three litters (n = 33) of a farm using the LAIV vaccine were selected for the study, a subset of them (n = 12) were left unvaccinated and a subset of piglets (n = 3) in vaccinated litters were also left unvaccinated to serve as sentinels. Selected piglets from the litters were sampled multiple days post vaccination (DPV) by collecting nasal swabs and blood, and were tested using a LAIV vaccine specific RT-PCR assay and hemagglutination inhibition assay against the LAIV strains respectively. Environmental specimens consisting of air and surface wipes were also collected. One hundred percent (21/21) of the vaccinated litters tested LAIV positive 1 DPV and until 6 DPV. In contrast, only five (5/33) of the thirty-three non-vaccinated pigs tested positive during the course of the study. Viable LAIV was confirmed in vaccinated pigs by cell culture and whole genome sequencing. In addition, low levels of LAIV RNA (RT-PCR Ct values ranging between 33 and 38) were detected in all air specimens collected on the day of vaccination and until 6 DPV (3/10). Pigs had maternally derived antibodies reactive against the LAIV strains which may have influenced the degree of shedding observed. Under the conditions of this study, shedding of the LAIV from vaccinated pigs was limited in time, resulted in minimal transmission to non-vaccinated pigs and was detected in low levels in aerosols collected in the vaccinated rooms likely influenced by the presence of maternally derived antibodies against the LAIV strains.

## Introduction

Influenza A virus (IAV) is an important cause of respiratory disease in pigs. IAV is a segmented RNA virus capable of sustaining high mutation rates (genetic drift) and gene reassortment (genetic shift) [1]. New reassortant viruses have the potential to result in emerging strains with new host ranges capable of producing severe disease and infections of pandemic potential [2]. IAV affects many hosts, including pigs and people, and the virus can be transmitted between species, making it a concern for both animal and public health [3].

In pigs, the respiratory disease caused by IAV is characterized by coughing, sneezing and fever with high morbidity but low mortality. IAV infections can result in decreased performance and increased antibiotic use [4]. IAV is endemic in pigs and co-circulation of strains within farms is common [5]. The main subtypes affecting pigs are H1N1, H1N2 and H3N2 and there is significant genetic diversity within and between these subtypes [6]. IAV can be transmitted by direct contact with secretions of infected animals or indirectly by aerosol and contaminated fomites [7]. Although direct contact between infected and susceptible pigs appears to be the main route of infection, several studies have demonstrated the presence of IAV in air collected inside and outside of farms and up to 1.6 km from an infected farm [8, 9]. Among the risk factors that facilitate IAV transmission and maintenance within herds are the introduction of replacement animals into breeding herds, the on-going birth of susceptible pigs within farrowing rooms, the continuous pig flow on growers and the implementation of low biosecurity measures [10, 11].

Vaccination against IAV is the main tool to control the disease [11–14]. Approximately 80% of large US sow farms report to vaccinate sows pre-farrow [15]. Moreover 38% of nurseries with more than 5,000 pigs have reported to vaccinate weaned pigs against IAV [15]. Pre-farrowing vaccination of pregnant sows aims to increase the transfer of antigen specific antibodies from the sow to the suckling pigs through colostrum [11], while vaccination of pigs aims to elicit an immune response in the pigs themselves. Until recently all the IAV vaccines available for use in pigs were virus inactivated vaccines, using whole cell virus or specific antigenic segments [16, 17]. This type of vaccine elicits mainly a humoral response with limited efficacy against heterologous viral challenges [13, 14]. Inactivated vaccines have shown to be effective at reducing transmission and clinical signs when the pigs are challenged with strains genetically similar to the vaccine strains [16, 18, 19]. However, the high mutation rates that RNA viruses sustain over time, and the on-going introduction of new strains into farms, limit the long-term efficacy of inactivated vaccines at controlling influenza. In contrast, live attenuated vaccines tend to result in broader immune responses by eliciting both, humoral and cellular immune responses [12] and some live attenuated vaccines also have the advantage of being effective in the presence of maternally derived antibodies as shown in previous studies [20].

Recently, a live attenuated virus vaccine (LAIV) (Ingelvac Provenza™, Boehringer Ingelheim Animal Health USA, Inc., Duluth, GA) became commercially available for use in pigs in the US [21]. Through reverse genetics, this vaccine contains two subtypes (H1N1 strain A/swine/Minnesota/37866/1999; MN99; and H3N2 strain A/swine/Texas/4199-2/1998; TX98) that have a truncation in the NS1 gene segment and is licensed for application in newborn pigs as early as 2 days of age. The NS1 protein has the major function of inhibiting the type 1 interferon mediated antiviral response from an infected cell. The truncation of the NS1 gene segment decreases the NS1 expression and the virus replication capacity [16]. The vaccine differs from other commercially available LAIV in humans because is not a cold-adapted attenuated LAIV. Nevertheless, the vaccine is live and can replicate in vaccinated animals.

Therefore, the objectives of this study were to assess the onset and duration of LAIV shedding in vaccinated pigs, investigate the transmission of LAIV from vaccinated to non-vaccinated pigs under field conditions and evaluate whether the LAIV could be aerosolized and detected in the environment.

## Materials and methods

### Ethics statement

This study was developed following the guidelines of the Institutional Animal Care and Use Committee (IACUC) from the University of Minnesota. The protocol was approved by the committee under protocol number 1806-36040A. The animals were owned by producers who provided written consent to have samples collected from the animals. At the conclusion of the study all the animals were weaned and transported to a nursery farm to continue growth until harvest, as dictated by the production company management practices.

### Experimental design

Five farrowing rooms of a 5,200 sow farm that vaccinated pigs at approximately 3–5 days of age with Ingelvac Provenza™ LAIV vaccine (Boehringer Ingelheim Animal Health USA Inc., Duluth, GA) were selected for the study. The sow herd had a history of IAV infection in weaned pigs and sows had been previously vaccinated with a whole inactivated autogenous IAV vaccine 4 weeks prior to farrowing. However, at the time of the study, the autogenous vaccine was not in use at the farm and piglets at weaning were IAV RT-PCR negative on 30 specimens collected monthly. The farm was considered stable to porcine reproductive and respiratory syndrome virus (PRRSV) based on the American Association of Swine Veterinarians PRRS herd classification [22] and *Mycoplasma hyopneumoniae* positive, two other common respiratory pathogens of pigs.

**Fig 1:**
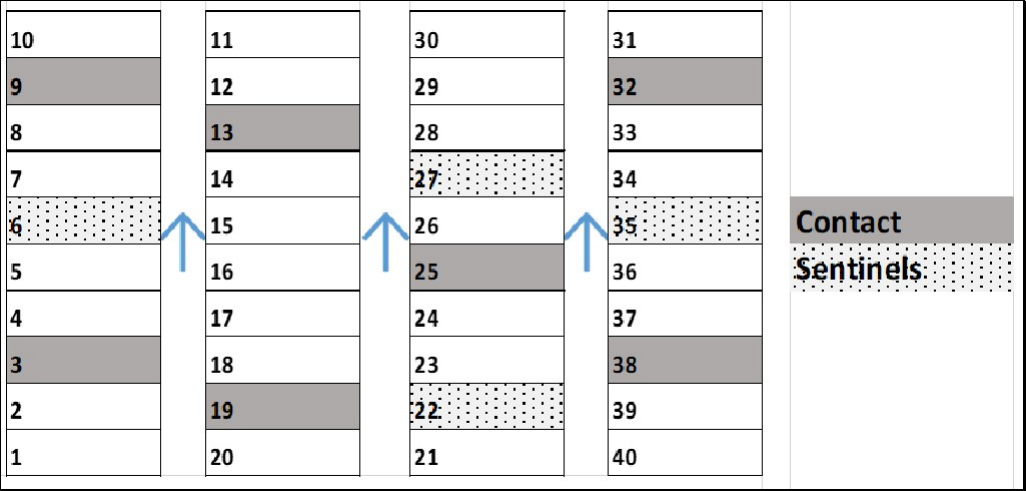
Diagram illustrating the distribution of litters within farrowing rooms. Dark grey rectangles represent the vaccinated contact litters. Light grey rectangles represent non-vaccinated sentinel litters. White rectangles represent vaccinated litters within the study room population but not part of the litters that were sampled. Arrows indicate the airflow in the farrowing room.

**Fig 2:**
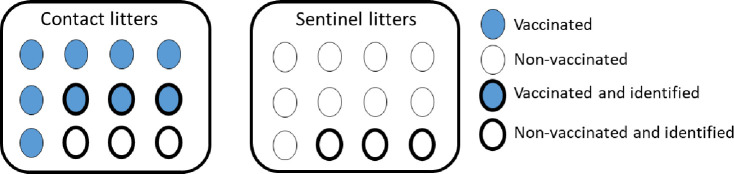
Description of the litters in the study. Blue circles represent the vaccinated pigs and white circles represent the non-vaccinated pigs. Circles with darker edges represent the pigs that were identified and selected for testing.

Thirty-three litters were conveniently selected within three of the 5 selected farrowing rooms following a pre-determined spatial pattern. Each room had capacity for a total of 40 litters out of which we selected 11 litters/room for the study (3 rooms x 11 litters = 33 study litters) (Fig 1). The selected litters had an average of 12 pigs per litter. In order to assess the shedding duration and the direct contact transmission of LAIV, 7 of the 11 litters within each room were assigned as direct contact litters. Within each vaccinated litter all pigs of the direct contact litters were vaccinated according to farm protocol with the exception of three pigs which did not receive vaccine so these three pigs could serve as unvaccinated contacts (Fig 2). In order to assess the indirect transmission of LAIV, 4 of the 11 litters in a room were assigned as sentinel litters. All the pigs on these sentinel litters were left unvaccinated. In total, there were 21 direct contact litters and 12 sentinel litters. Within each contact litter, three vaccinated and three non-vaccinated pigs were individually identified with a numbered ear tag. Within the sentinel litters, three pigs were also identified with a numbered ear tag. Once the study started and litters were selected, there were no additional pigs from other sows added to the litters.

The air was sampled after vaccination in the three farrowing rooms containing the study litters and two other adjacent farrowing rooms also containing vaccinated pigs. All five farrowing rooms were sampled after vaccination to assess aerosolization of LAIV.

Pigs were intranasally vaccinated at 3–5 days of life. Pigs were held by a worker, securing the head and pointing the snout upwards while introducing the syringe in one of the nostrils and administering 1 dose of 1 ml of the vaccine in spray form following manufacturer recommendations [21].

### Sampling protocol and specimen collection

In order to determine the IAV status of the herd prior to the start of the study, 14 days prior to vaccination (Day -14), thirty oropharyngeal swabs were obtained from pigs of weaning age (~18 days), using rayon-tipped swab applicators with Stuart’s medium (BBL CultureSwab™ liquid Stuart single plastic applicator; Becton, Dickinson and Com. Sparks, MD, USA). Collection of samples from the oldest pigs prior to weaning is considered a sensitive method to detect IAV in endemic swine herds [23].

**Fig 3:**
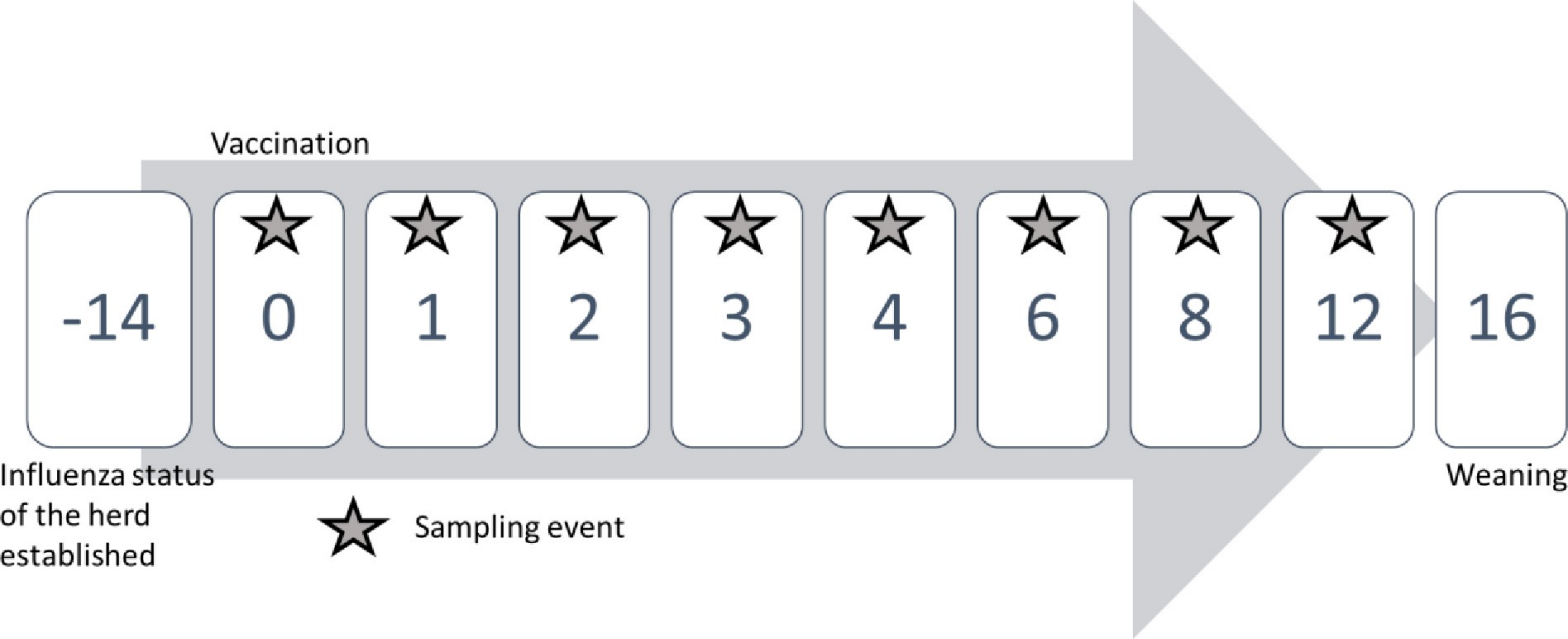
Study timeline. Numbers represent the days of the study in reference to when vaccination took place. Stars represent when sampling events which included the collection of nasal swabs, udder skin wipes, surface wipes and collection of environmental specimens took place.

To assess LAIV shedding and transmission, nasal swabs were obtained from the individually identified pigs at day 0, 1, 2, 3, 4, 6, 8 and 12 days post-vaccination (DPV) using a mini-tip Pur-Flock Ultra® swab (Puritan Medical Products LLC, Guilford, Maine, USA) (Fig 3). Skin wipes from the udder of lactating sows of the study and surface wipes of the farrowing crates from those same litters were collected at day 0, 1, 2, 3, 4, 6, 8 and 12 DPV, using a 3 x 3 inches sterile gauze impregnated with 10 ml of DMEM-Dulbecco’s Modified Eagle Medium Gibco™(Grand Island, NY, USA) supplemented with 0.5 ml of Gentamicin Sulfate (BioWhittaker®, Walkersville, MD USA) and 5 ml of antibiotics and antimycotic, Anti-Anti (100x) Gibco™(Grand Island, NY, USA). Udder wipes were used because they collect oral and nasal secretions deposited on the udder skin of the sow by piglets during suckling and reflect the IAV status of the piglets at sampling. Both of these methods, udder wipes and surface wipes, can be used effectively for the detection of IAV in pig farms [23].

After vaccination was completed (0 DPV), air was collected from the room immediately and at 1, 2, 3, 4, 6 and 8 DPV. Air was collected for 30 min using an air cyclonic sampler (Midwest Micro-Tek, Brookings, SD, USA) capable of collecting 200 L/min. Ten ml of DMEM-Dulbecco’s Modified Eagle Medium Gibco™(Grand Island, NY, USA) supplemented with 0.5 ml of Gentamicin Sulfate (BioWhittaker®, Walkersville, MD USA) and 5 ml of antibiotics and antimycotic, Anti-Anti (100x) Gibco™(Grand Island, NY, USA) were added to the air collection vessel and after collection, the liquid in the collection vessel was transferred into a tube, and transported to the laboratory under refrigerated conditions.

Specimens from the farrowing room environment, specifically the airborne particles that settled on surfaces, were also collected immediately after completion of the vaccinations at 0 DPV and in each room and at 1, 2, 3, 4, 6 and 8 DPV. These specimens were collected using 6 pieces of aluminum foil paper, 1 meter long each, located on top of farrowing crates for 60 min [23]. The deposited particles were collected by wiping the foil paper surface with a 3 x 3 inches sterile gauze impregnated with DMEM-Dulbecco’s Modified Eagle Medium Gibco™(Grand Island, NY, USA) supplemented with 0.5 ml of Gentamicin Sulfate (BioWhittaker®, Walkersville, MD USA) and 5 ml of antibiotics and antimycotic, Anti-Anti (100x) Gibco™(Grand Island, NY, USA).

Blood was collected from all individually identified pigs before vaccination at day 0 and at 15 DPV by venipuncture using BD Vacutainer® (Becton, Dickinson and Company, Franklin Lakes, NJ, USA) to detect IAV antibodies.

All specimens were transported refrigerated at 4˚C to a University of Minnesota research laboratory.

### Influenza A virus detection assays

Piglet oropharyngeal swabs collected 14 days prior to the start of the study and piglet nasal swabs collected at day 0 were tested by an IAV RT-PCR targeting the conserved matrix gene using previously described procedures [24].

Nasal swabs collected on days 0 and at 1, 2, 3, 4, 6, 8 and 12 DPV were pooled within litters, by vaccination status, and then tested as a pool of three swabs. Udder skin wipes, surface wipes, air and particle deposition wipes were tested individually. All these specimens were tested for IAV using reagents for vaccine specific RT-PCR (Vaccine-PCR reagents) [25] (samples collected at 0 DPV were tested using both RT-PCR types). Briefly, each vaccine-PCR reaction consisted of 25 μL total volume using Path-ID™ Multiplex One-Step RT-PCR kit® by Ambion® (Foster City, California, USA), containing 0.1 mL nuclease-free H_2_O, 0.5 μL 25 μM forward primer, 0.4 μL 25 μM reverse primer, 1.00 μL 12.5 μM probe, 2.5 μL Enzyme, 12.5 μL 2X RT Buffer. Using a MicroAmp® Fast Optical 96 well reaction plate by Applied Biosystems (Foster city, California, USA) on a 7500 Fast Real-Time PCR System by Applied Biosystems (Foster City, California, USA), with the following conditions, 45˚C for 10 minutes, 95˚C for 10 minutes, 40 cycles of 97˚C for 2 seconds and 60˚C for 40 seconds, and cooling at 40 ˚C for 20 seconds. Specimens with cycle threshold (Ct) values < 38 were considered positive and Ct ≥ 38 considered negative.

The specimens that tested vaccine RT-PCR positive were selected for virus isolation using Madin–Darby Canine Kidney (MDCK) cells as described previously [26]. Viral isolates were further submitted for whole genome sequencing to verify presence of the vaccine LAIV strain. Briefly, the cell culture suspension IAV isolates were processed by the University of Minnesota veterinary diagnostic laboratory (MVDL) for total nucleic acid using QIAamp MinElute Virus Spin Kit (Qiagen) following the manufacturer’s instruction. The purified total nucleic acid samples were processed for cDNA synthesis and library preparation using the SMARTer® Stranded Total RNA-Seq Kit v2, according to manufacturer instructions (Clontech, a Takara Bio Company, CA, USA). Final pooled libraries were submitted to the University of Minnesota Genomics Center for 150 bp paired-end cycle sequencing run on the Illumina MiSeq System (Illumina, San Diego, CA). After automated cluster generation, the genomic sequence reads (FASTQ) files were obtained for further analysis using bioinformatics pipeline developed at the MVDL including trimming, denovo assembly and fasta sequences of assembled genome. Genomic sequence reads (FASTQ) files of each IAV gene segment were uploaded to the NCBI database to identify reference strains with the highest percent nucleotide identity with the study strains.

### Influenza A virus antibody detection assays

The collected blood was allowed to clot, the serum was separated from the clot, and the sera was tested using a blocking enzyme-linked immunosorbent assay (ELISA) assay (IDEXX Swine influenza Virus Ab test; IDEXX Laboratories, Inc.) that measures antibodies against the nucleoprotein (NP) of IAV as described previously [27]. Sera samples were considered positive for IAV antibodies if the sample to negative (S/N) ratio was ≤0.6 and an S/N ratio >0.6 was considered negative for IAV antibodies per the ELISA manufacturer’s guidelines.

Sera to detect maternally derived cross-reactive antibodies against the LAIV strains were also tested using an hemagglutination inhibition (HI) assay following methods by the World Health Organization [28]. Briefly, serum from 1 pig per litter (n = 33) was first treated with three parts receptor destroying enzyme (RDE; Denka Seiken, Tokyo, Japan) for 18h at 37˚C, then it was heat inactivated at 56˚C for 30 min. Turkey red blood cells at 20% concentration were added to remove nonspecific hemagglutinin inhibitors and natural serum agglutinins. The HI assay was performed using turkey red blood cells and antibodies were assessed against 2 LAIV like strains as antigens. To perform the H1 HI, a field isolate obtained from the University of Minnesota Veterinary Diagnostic Laboratory with >99% homology to the H1 segment of the LAIV strain (H1N1 A/swine/Minnesota/37866/1999) was used. To perform the H3 HI we used the H3N2 A/swine/Texas/4199-2/1998 strain which is the predecessor of the H3 isolate contained in the LAIV.

### Statistical analysis

The cycle threshold (Ct) results obtained from the vaccine RT-PCR were compared by specimen type and vaccination status using an ANOVA test and Welch two-sample t-test. The S/N results obtained from the ELISA test were compared by vaccination status using a paired Student’s t-test. The HI titers obtained were log2 transformed and compared by vaccination status using a Welch two-sample t-test. P-values <0.05 were considered statistically significant. All these analyses were carried out in R version 3.3.3 (R Core Team, 2013).

## Results

### RT-PCR

Oropharyngeal swabs from pigs of weaning age collected 2 weeks prior to initiate the study and nasal swabs collected at day 0 were all negative by RT-PCR targeting the conserved matrix gene of IAV.

At 1 DPV, 100% (21/21) of the nasal swabs from the vaccinated pigs were IAV positive by vaccine RT-PCR. The number of IAV positive litters decreased daily and the last positive vaccine RT-PCR was detected at 6 DPV (2/21 litters) (Table 1). The direct contact non-vaccinated pigs tested IAV positive at 1 DPV (2/21) and 4 DPV (1/21). Sentinel litters indicative of indirect contact transmission tested IAV positive at 2 DPV (1/12) and 12 DPV (1/12) (Table 1).

**Table 1:** Number of influenza A virus vaccine-specific RT-PCR positive pools of nasal swabs detected in litters by day, vaccination status and type of contact.

	Days post vaccination							
Litters	0^&^	1	2	3	4	6	8	12
**Vaccinated**	0/21*	21/21	14/21	9/21	5/21	2/21	0/21	0/21
**Unvaccinated direct contact**	0/21	2/21	0/21	0/21	1/21	0/21	0/21	0/21
**Unvaccinated indirect contact (sentinels)**	0/12	0/12	1/12	0/12	0/12	0/12	0/12	1/12

^&^Samples collected at 0DPV were tested using both the matrix RT-PCR and the vaccine RT-PCR.

*Number of positive litters / total number of litters tested.

Nasal swabs were tested in pools of three. Swabs within a litter and of the same vaccination status were pooled together. A specimen was considered positive when the vaccine RT-PCR cycle threshold value was <38.

Seventy-six percent (16/21) of the udder skin wipes collected from sows with vaccinated litters tested positive at 1 DPV and positive udder skin wipes were detected until 12 DPV (1/21). Eight percent (1/12) of udder skin wipes collected from sows with non-vaccinated sentinels tested positive at two sampling points, 6 and 12 DPV (Table 2).

**Table 2:** Number of influenza A virus vaccine-specific RT-PCR positive litters detected in udder skin wipes by vaccination status and days post vaccination.

	Days post vaccination							
Litters	0	1	2	3	4	6	8	12
**Vaccinated**	0/21*	16/21	6/21	3/21	5/21	2/21	0/21	1/21
**Non-vaccinated (sentinels)**	0/12	0/12	0/12	0/12	0/12	1/12	0/12	1/21

*Number of positive litters / total number of litters.

Specimens were tested individually. A specimen was considered positive when the cycle threshold value was < 38.

Of the farrowing crate surface wipes, those crates holding vaccinated litters were IAV positive by vaccine RT-PCR at 1 DPV (47%) and until 8 DPV (4%). One crate (1/12) surface wipe that held non-vaccinated (sentinel) pigs was IAV positive at 1 DPV (Table 3).

**Table 3:** Number of influenza A virus vaccine-specific RT-PCR positive litters detected in surface wipes by vaccination status and time.

	Days post vaccination							
Litters	0	1	2	3	4	6	8	12
**Vaccinated**	0/21*	10/21	5/21	2/21	2/21	0/21	1/21	0/21
**Non-vaccinated (sentinels)**	0/12	1/12	0/12	0/12	0/12	0/12	0/12	0/21

*Number of positive litters / total number of litters.

Specimens were tested individually. A specimen was considered positive when the cycle threshold value was < 38.

LAIV was detected by vaccine RT-PCR in forty-six percent (25/54) of the total air specimens collected. Detection immediately after vaccination was 100% and decreased until 6 DPV when the last positive specimens were detected (Table 4). The Ct values ranged from 33 to 39.

**Table 4:** Number of influenza A virus vaccine-specific RT-PCR positive environmental specimens including air and deposited airborne particles by days post vaccination.

	Days post vaccination						
Sample type	0	1	2	3	4	6	8
**Air**	8/8*	5/8	3/6	4/10	2/6	3/10	0/6
**Particle deposition**	17/25	6/24	2/18	5/30	2/18	6/30	1/18

*Number of positive specimens/total number of specimens.

Sixty-eight percent (17/25) of the specimens containing deposited airborne particles were IAV positive by vaccine RT-PCR on the day of vaccination. Detection of IAV positive specimens decreased gradually although positive specimens were detected at all sampling days (Table 4).

**Fig 4:**
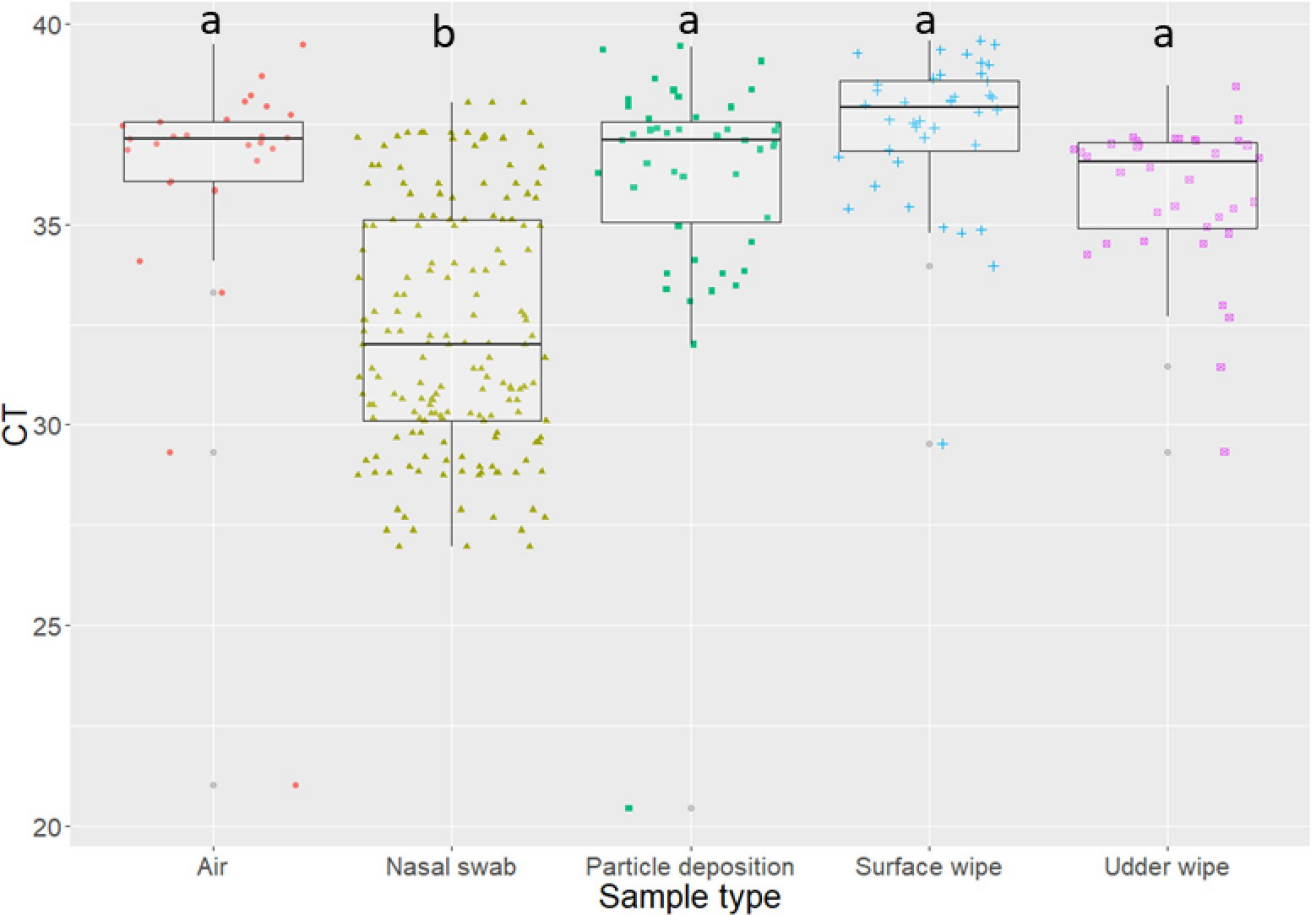
Distribution of influenza A virus cycle threshold (Ct) values obtained by vaccine RT-PCR by specimen type. Letters represent statistical difference between specimen types at p<0.05.

The cycle threshold (Ct) values obtained by specimen type are shown in Fig 4. The nasal swabs had the lowest Ct values. Since the vaccine RT-PCR is semi-quantitative, lower Ct values are suggestive of more viral RNA present and results obtained from the nasal swabs were significantly lower than the ones obtained by the other sampling methods (P value = 0.0001). In general, all environmental specimens had high Ct values suggestive of low viral RNA quantity; however, there was no statistical difference in the Ct values obtained among environmental specimens.

**Fig 5:**
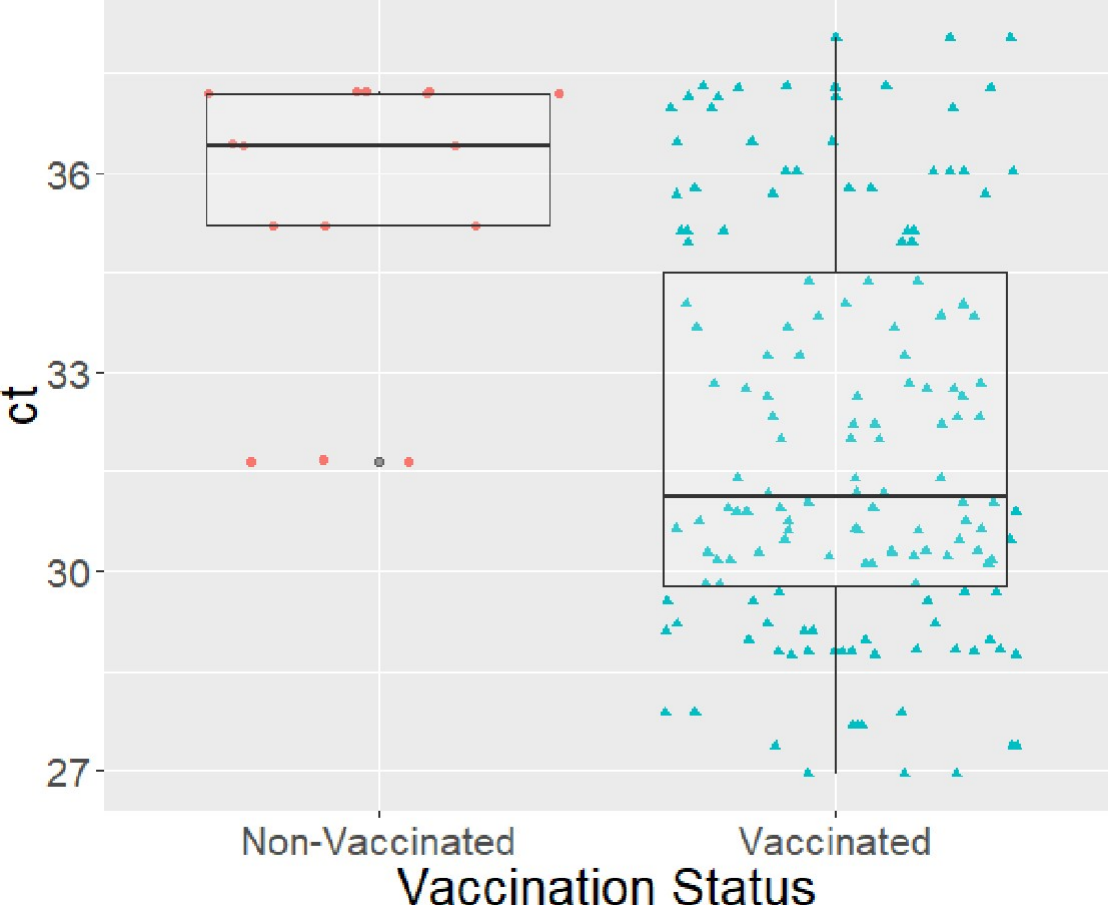
Distribution of influenza A virus cycle threshold (Ct) values obtained by vaccine RT-PCR from nasal swabs by vaccination status.

The Ct values obtained from the vaccine-RT PCR tests of the nasal swabs are shown in Fig 5. The nasal swabs from vaccinated pigs had significantly lower Ct values than non-vaccinated pigs (P value = 0.0001).

### Virus isolation and whole genome sequencing

LAIV was isolated in 11% of the specimens cultured (4/36). The 4 specimens that contained viable virus were from nasal swabs of vaccinated pigs at 1 and 3 DPV. The isolates were confirmed to be vaccine virus by testing positive with the vaccine specific RT-PCR and by whole genome sequencing. Table 5 shows the percent homology of each of the isolates compared to all gene segments of the LAIV reference strains (GenBank accession numbers are shown in the table). For all the isolates and all segments the LAIV parental strain, A/swine/Texas/4199-2/1998(H3N2) was the strain with the highest percentage of nucleotide identity.

**Table 5:** List of viruses with the highest sequence identity to each gene from the sequenced isolates using virus sequences available in GenBank.

Gene	Positions	Virus with highest percentage of nucleotide identity	Accession numbers	Homology (%)	Homology (%)	Homology (%)	Homology (%)
				Isolate 1	Isolate 2	Isolate 3	Isolate 4
PB2	1–2280	A/swine/Texas/4199-2/1998(H3N2)	CY095672.1	100	100	100	100
PB1	1–2277	A/swine/Texas/4199-2/1998(H3N2)	CY095673.1	100	100	100	100
PA	1–2151	A/swine/Texas/4199-2/1998(H3N2)	CY095674.1	99.95	100	100	100
HA	1–1701	A/swine/Texas/4199-2/1998(H3N2)	CY095675.1	99.94	99.94	99.94	99.94
NP	1–1497	A/swine/Texas/4199-2/1998(H3N2)	CY095676.1	100	100	100	100
NA	1–1410	A/swine/Texas/4199-2/1998(H3N2)	CY095677.1	99.93	99.93	99.93	99.93
M	1–982	A/swine/Texas/4199-2/1998(H3N2)	CY095678.1	99.90	100	100	100
NS	1–838	A/swine/Texas/4199-2/1998(H3N2)	CY095679.1	92.67	92.67	92.67	92.67

### Serology

**Fig 6:**
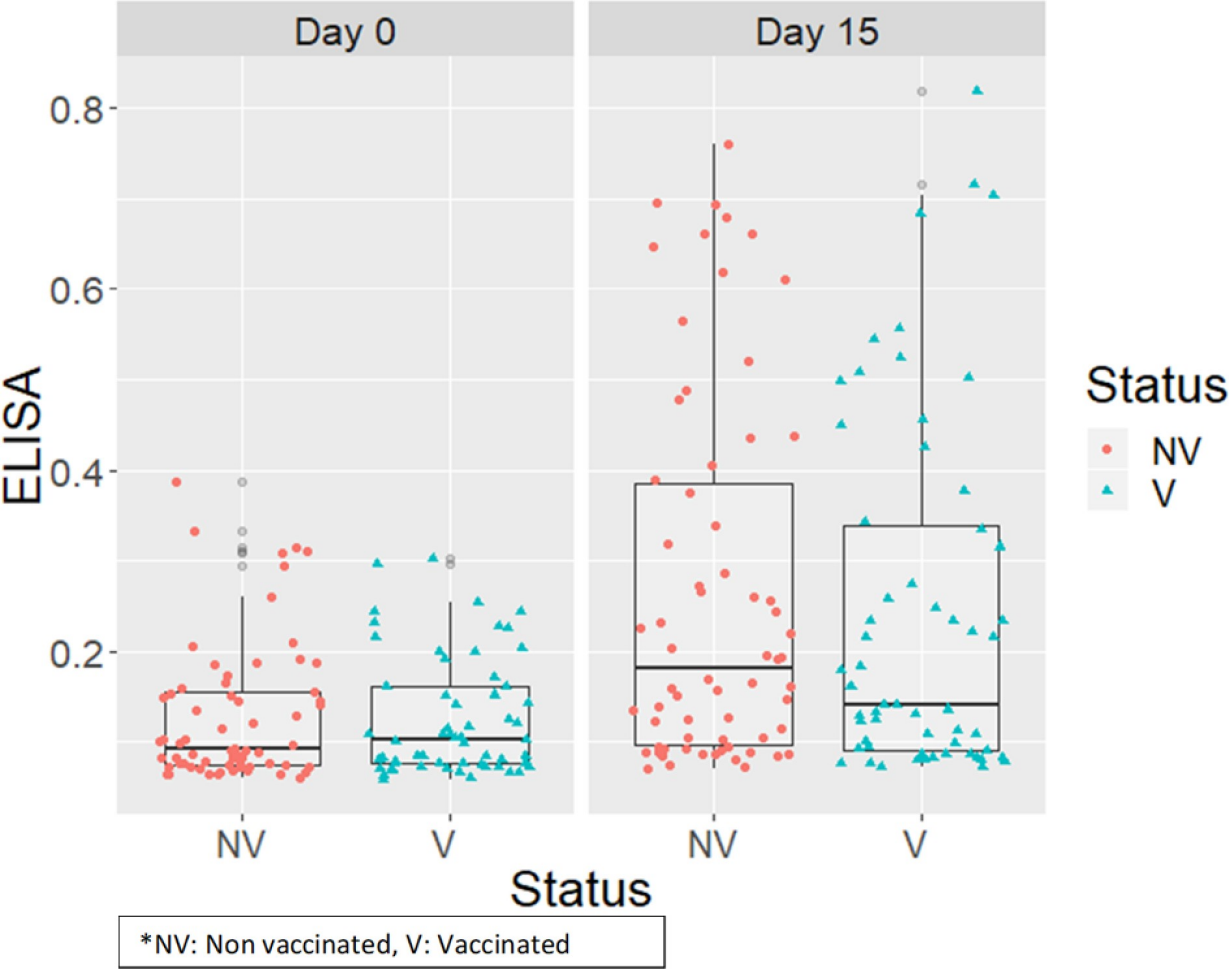
Distribution of sample to negative (S/N) ratio values obtained by ELISA test in sera samples before vaccination (day 0) and at 15 days post vaccination in vaccinated and non-vaccinated pigs.

Before vaccination, at 0 DPV when the pigs were 3 days of age, all pigs had antibodies of maternal origin against IAV as detected by the ELISA test. At 18 days of age (15 DPV), 93% and 86% of the pigs from vaccinated and non-vaccinated groups respectively were still serum antibody positive for IAV by the ELISA test, but with more pigs having S/N values approaching the negative cutoff value. There was no statistically significant difference between the two groups at 18 days of age (P value = 0.56) (Fig 6).

**Fig 7:**
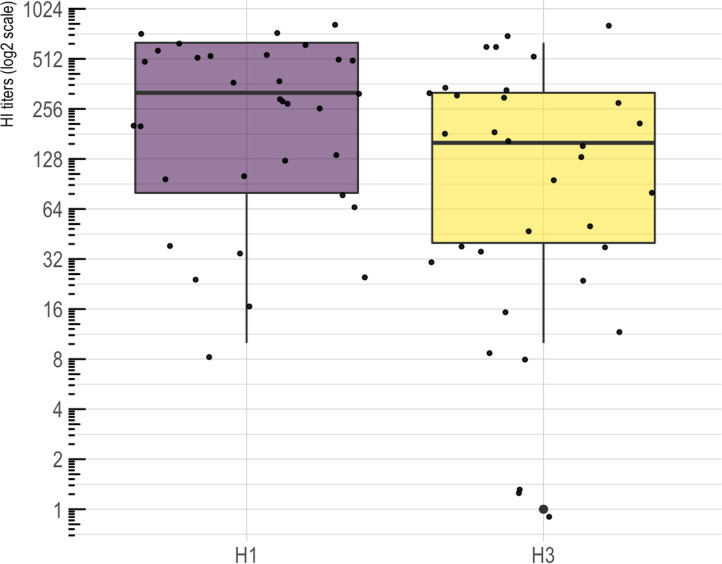
Distribution of titers against H1 and H3 isolates obtained by HI assay on sera samples from piglets before vaccination (day 0).

Results from the HI assay at 0 DPV indicated presence of cross reactive antibodies against LAIV strains. The distribution of the HI titers against H1 and H3 LAIV strains are shown on Fig 7. For H1, pigs had a geometrical mean titer of 197.39 with 3.48 of standard deviation. For H3, pigs had a geometrical mean titer of 72.07 with 6.36 of standard deviation. There were no statistical significant differences in HI titers between vaccinated and not vaccinated litters (results not shown).

## Discussion

One of the benefits attributed to LAIV vaccines is the broad immune responses they induce. Broad immunity is usually associated with broad cross-protection against genetically diverse strains circulating in pigs[16]. However, there are concerns about the safety and transmission of LAIV in pigs because IAV frequently reassort and mutate. In this study, we found that shedding of a newly commercialized LAIV vaccine (Ingelvac Provenza^TM^) in commercial pigs and the environment was possible but it was limited in duration and resulted in minimal transmission to non-vaccinated pigs in direct contact or to non-vaccinated sentinel pigs with indirect-contact within the same air space. The pigs in the study had maternally derived antibodies that cross-reacted with the LAIV strains which likely influenced the degree of LAIV shedding and transmission observed.

Shortly after LAIV administration, LAIV was detected in the vaccinated pigs which was expected since the vaccine virus is live and capable of replication despite being attenuated. LAIV was also detected for up to 6 days post-vaccination, and duration of LAIV shedding in this study was similar to other studies with this vaccine under experimental conditions [29] and mimics the duration of shedding described for pigs challenged with wild-type IAV in IAV inoculation studies [30]. Furthermore, we were able to isolate LAIV from vaccinated pigs indicating presence of replicating virus and we confirmed through whole genome analysis that all the IAV isolates recovered during the study were of LAIV origin. There was no evidence of reassortment between LAIV and other endemic influenza viruses.

At 3 days of age all pigs had antibodies against IAV. These were maternally derived antibodies (MDA) since pigs are unable to develop antibodies against IAV within the first few days of life. It is likely that LAIV shedding was influenced by the presence of maternally derived antibodies given that pigs had relatively high levels of cross-reactive antibodies against LAIV parental strains. MDAs are known to reduce shedding and transmission of IAV [19] but the magnitude of the reduction in transmission depends on the degree of cross-reactivity between the circulating IAV strains [20], the titer levels [31] and avidity of the antibodies [32]. Results in LAIV shedding may differ between herds based on the levels of MDA present at the farm. The reactivity of antibodies against the LAIV was somewhat unexpected since the LAIV parental strains had been isolated in the late 90’s. However, although IAV vaccination in sows was not conducted at the time of the study, the farm had a history of using an autogenous multivalent vaccine which may explain the origin of these antibodies. Unfortunately, we do not have additional information on which strains were included in the autogenous vaccine and investigating the cross-reactivity of the autogenous vaccine with the LAIV was outside the scope of this study.

Furthermore, MDA levels decreased throughout the study as evidenced by the serological results obtained in pigs at weaning. Lower levels of antibodies at weaning may increase the pig’s susceptibility to infection and facilitate LAIV transmission as the pigs become older. This latter point could not be evaluated in this study since the study terminated at weaning. Further research is needed to evaluate transmission of LAIV in older pigs or pigs with low or no levels of MDA immunity.

Since one of the main concerns of LAIV is the transmission to susceptible hosts, we were especially interested in evaluating shedding of LAIV to direct contact non-vaccinated pigs within a litter. We made sure that the litter remained intact throughout the study with plenty of interaction between the pigs within the litter to facilitate contact between vaccinated and non-vaccinated pigs. In this study we showed that LAIV transmission from vaccinated to non-vaccinated pigs was possible but limited in time and scope since IAV was detected in direct contact non-vaccinated pigs in only three litters at 1 DPV and 4 DPV. These were the only positive sampling events that took place between vaccination and weaning, a timespan of approximately 21 days. Given that the detection in non-vaccinated contact pigs was sporadic and that we could not obtain viable virus by cell culture from these vaccine-specific RT-PCR positive specimens with Ct values near the 38 cutoff for positivity, we cannot be certain that the virus did indeed replicate in the non-vaccinated contact pigs. It is possible that pigs were protected against LAIV due to the presence of MDA, or only transiently positive due to deposition of inactivated virus in the mouth during suckling or in the nostrils from inhalation of airborne LAIV. Results from the udder skin wipes were also positive on those same days these 3 contact litters tested vaccine RT-PCR positive, suggesting that vaccinated pigs could serve as a source of contamination of the udder skin which in turn could serve as a source of exposure or contamination to non-vaccinated pigs. However, it is more likely that the results from non-vaccinated pigs reflected detection of inactivated virus rather than an active infection, and/or the ability of the MDA to prevent infection in the direct contact pigs.

Transmission to non-vaccinated sentinel pigs having indirect contact with LAIV-vaccine was also minimal. Only 2 sentinel litters tested positive during the course of the study. The positive specimens were from nasal swabs collected at 2 and 12 DPV. The sentinel pigs that tested positive at 2 DPV did not test positive in the following sampling points which could suggest lack of virus replication in the sentinel pigs. However, the sample at 12 DPV was obtained on the last sampling day of the study and thus we cannot conclude that replication of the vaccine virus did not occur thereafter. Nevertheless, the udder skin wipes and surface wipes from these two sentinel litters were all negative on these particular days and we did not pursue virus isolation due to the high Ct values observed which is usually indicative of the presence of low quantities of viral RNA in the specimens. Furthermore, we cannot rule out airborne exposure of the sentinel pigs given that LAIV was detected in the air throughout the study.

There was occasional detection of IAV by vaccine RT-PCR in udder skin and surface crate wipes collected from vaccinated and non-vaccinated litters even when the nasal swabs tested negative. We speculate that this finding may possibly be just environmental contamination due to airborne particle settling on surfaces that later find their way in the piglet’s oral secretions.

LAIV was detected by vaccine RT-PCR in the air in approximately half of the specimens collected. We detected viral RNA in air on all days following vaccination except on the last sampling day (6 DPV). We observed similar results with the wipes of the settled airborne particles on surfaces within the farrowing rooms. Most (n = 23/163) of the positive specimens, however, were collected immediately after vaccination which likely represents LAIV airborne dispersion during the vaccine administration process or early post-vaccination virus replication. Though detection of LAIV in the environmental specimens was possible, transmission to non-vaccinated sentinel pigs that were in the same air space was not always evident. The majority of the Ct values obtained in the environmental specimens were near the 38 cutoff value which indicates that the amount of viral RNA in the air was low. Conversely, we cannot rule out that infection with LAIV occurred due to airborne exposure to non-vaccinated pigs. It is important to point out that each farrowing room had 40 farrowing crates from which only 11 were enrolled in the study. The remaining 29 crates were vaccinated as dictated by farm protocol. Therefore, there was a high proportion of pigs vaccinated in the same air space, which could be the reason for the multiple positive specimens detected in the environment.

All pigs in the study showed a decay in the antibody levels detected between vaccination and weaning which suggests a natural decline of maternally derived antibodies, but it also indicates a lack of humoral response to this LAIV. This lack of seroconversion coincides with findings from other studies with LAIV vaccine where no humoral response was detected after vaccination [13].

The vaccine strain specific reagents used for the vaccine RT-PCR that was used to test the collected specimens has an acceptable sensitivity and specificity. It targets a signature sequence within the truncated portion of the NS1 gene that is specific for the LAIV and not found in field viruses [25]. It is important to note that it is possible for the LAIV to accumulate additional mutations in the NS1 gene that may fail to be detected by the vaccine RT-PCR reagents. However, we did not have any evidence of mutations in the NS1 gene that may result in failure of detection by the vaccine-specific RT-PCR, occurred, likely due to the short duration of the study. Also, we confirmed by whole genome sequencing that all the isolates recovered during the study were of LAIV origin which supports the use of this vaccine specific RT-PCR.

Despite our findings of limited shedding from vaccinated pigs to non-vaccinated pigs with direct contact and sentinels with indirect contact, it is important to emphasize the need to use LAIV when there are no endemic swine IAV viruses circulating in the population. Reassortment between endemic swine IAV and LAIV has been documented [33] and it is possible that reassortment could still take place in situations of limited LAIV shedding. Nevertheless, efforts need to be made to minimize presence of endemic IAV viruses at time of vaccination with LAIV.

In summary, in this study we found evidence of limited transmission of LAIV to non-vaccinated, sentinel pigs with indirect contact to vaccinates but sharing the same air space and to non-vaccinated pigs in direct contact with vaccinated pigs. We concluded based on our results, that although some LAIV was detected in non-vaccinated pigs with, either direct or indirect contact, LAIV detection did not result in sustained shedding in the non-vaccinated pigs likely influenced by the presence of MDA cross-reactive against the LAIV strains. More research is needed to further understand the transmission dynamics of LAIV, the impact of MDA on LAIV transmission and the implications that the use of LAIV could have on influenza control in swine farms.
